# Determination of a ‘point of no return’ in refractory chronic subdural hematomas: A case report and review of the literature

**DOI:** 10.3892/mi.2024.199

**Published:** 2024-10-10

**Authors:** Alexandros G. Brotis, George Fotakopoulos, Vasiliki Epameinondas Georgakopoulou, Adamantios Kalogeras, Theodosis Spiliotopoulos, Ioannis Ioannidis, Kostas N. Fountas

**Affiliations:** 1 Department of Neurosurgery, General University Hospital of Larissa, 41221 Larissa, Greece; 2 Department of Pathophysiology, Laiko General Hospital, Medical School, National and Kapodistrian University of Athens, 11527 Athens, Greece; 3 Department of Radiology, Larissa University Hospital, Faculty of Medicine, School of Health Sciences, University of Thessaly, 41221 Larissa, Greece

**Keywords:** chronic subdural hematoma, recurrence, treatment, intraparenchymal hemorrhage, neurosurgery

## Abstract

Recurrence following the surgical evacuation of a chronic subdural hematoma (CSDH) occurs in up to 33% of cases. Several clinical and radiologic factors have been identified that are associated with the recurrence of hematoma. However, the optimal treatment for recurrent CSDH has not yet been determined. The present study, based on a case report, reviews the predictors and treatment options for refractory CSDHs. An 85-year-old male patient presented with a symptomatic bilateral CSDH. The hematoma was initially removed through a burr hole and closed drainage system, resulting in clinical improvement and in the radiographic resolution of the hematoma. At the first recurrence, steroids were administered and the hematoma was re-evacuated. After 1 month, the patient returned comatose due to a massive right subdural hematoma and was treated with an ipsilateral craniotomy and a membranectomy. After 2 days, the patient succumbed due to massive intraparenchymal bleeding. The treatment of refractory CSDHs is challenging. The failure of brain re-expansion and senile atrophy appear to be the key predictors of recurrence. Patients who are at high-risk need to be identified promptly and treated with a multidisciplinary approach that considers adjuvant medications, middle meningeal artery embolization and repeat hematoma evacuation, probably with a membranectomy and an endoscope.

## Introduction

The outcome of surgically managed chronic subdural hematoma (CSDH) is usually improved, with a median surgical intervention-to-resolution time achieved until 160 days (interquartile range, 85-365 days) (1-3). However, the reappearance of hematoma occurs in up to 33% of patients, which is related to higher morbidity and mortality rates (4-10). The primary pathogenetic mechanisms of the condition remain uncertain; however, there is evidence of an interaction connecting inflammatory, fibrinolytic and angiogenic pathways (2,3). Numerous studies have recognized possible clinical, radiological and surgical risk factors for the recurrence of hematomas, including an advanced age, the male sex and other characteristics inherent to the hematoma (4-6,10-12). In extremely old patients suffering from multiple major comorbidities, there are extensive repercussions, suggesting that those at an older age are at a higher risk of developing recurrent hematomas (4). A variety of neurological symptoms frequently arise, ranging from mild focal symptoms related to the long tracts to coma and mortality (2,3). The diagnosis is typically complete with a computed tomography (CT) scan of the head, which illustrates the hematoma and provides brain compression (2,3).

Surgical hematoma evacuation constitutes the gold standard for the recurrence of CSDH (2,3,13,14). Drugs such as steroids, statins and tranexamic acid have been used as adjunctive therapy to diminish the risk of reappearance. Εqually, middle meningeal artery embolization (MMAE) recently gave hopeful outcomes in recurrent cases (13,15-19). Neuroedoscopy helps determine the adhesions in compartmentalized lesions (20,21), while the role of membranectomy remains to be established (22).

The present study describes the case of an elderly male patient with refractory CSDH who was treated on an escalated basis. In addition, after reviewing the relevant literature, the complexity of refractory CSDH and all major available treatment alternatives are discussed. Finally, the present study attempted to identify the patient's ‘point of no return’, if any.

## Case report

**Figure 1:**
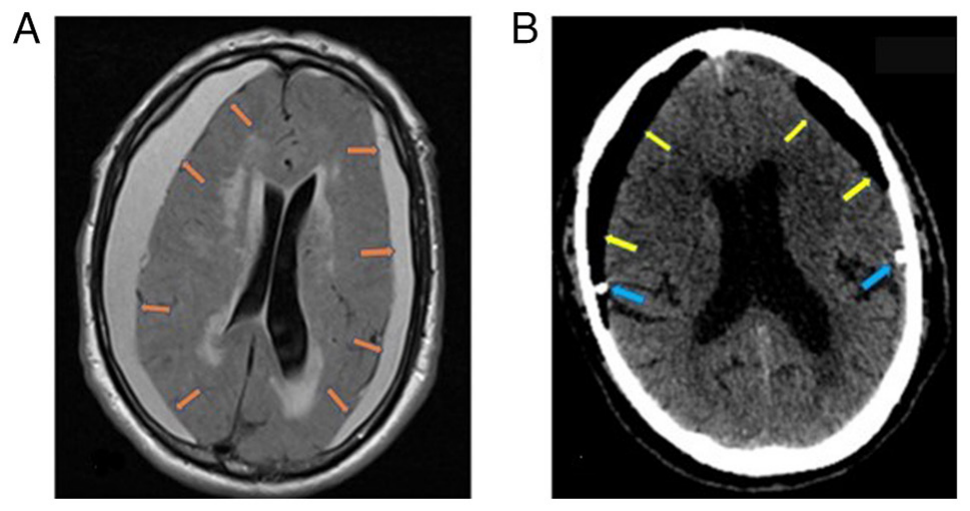
(A) A brain MRI revealed bilateral chronic subdural hematomas. The orange arrows indicate the brain-to-hematoma interface. (B) The post-operative computed tomography scan revealed that the hematoma was completely evacuated, and the cavity (yellow arrows) was filled with air as the brain failed to re-expand. The draining catheters (blue arrows), connected to a closed collection system, are in place.

An 85-year-old male patient presented to the University Hospital of Larissa (Larissa, Greece) in April, 2023 for the first time complaining of increasing headaches and instability while walking. The medical records of the patient mentioned anticoagulant therapy (clopidogrel, 75 mg per day) for stroke prevention by atrial arrhythmias (propafenone, 150 mg per day) and diabetes type II (gliclazide, 60 mg per day). A clinical examination revealed mild left hemiparesis [4/5 muscle strength according to the Medical Research Council (MRC) Scale for Muscle Strength], slow thinking ability, and mild disorientation. Magnetic resonance imaging (MRI) of the brain reealed bilateral subdural hematomas that compressed the brain parenchyma (Fig. 1A). The patient underwent hematoma evacuation through burr holes and a closed drainage system. The post-operative head CT scan revealed complete hematoma evacuation, and on the 2nd post-operative day, the patient could walk unassisted without any neurological deficit (Fig. 1B).

**Figure 2:**
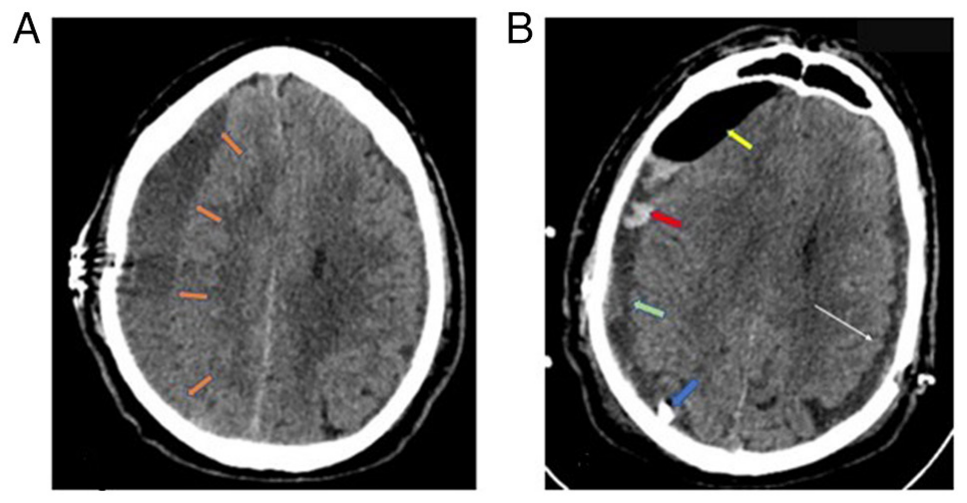
(A) At 2 weeks after the initial hematoma evacuation, the patient returned with recurrence of the right-side hematoma (orange arrows). (B) The post-operative computed tomography scan revealed partial hematoma evacuation (green arrow) with acute hemorrhage on a top of chronic subdural hematoma (red arrow) and air (yellow arrow) in the subdural space. The blue arrow indicates the draining catheter, whereas the white arrow points to the recurrence of the left-side hematoma.

After 2 weeks, the patient returned to the hospital with a recurrence of symptoms and a Glasgow Coma Scale (GCS) score of 13/15 (M:5, V:5, E:3), left hemiparesis and profound disorientation. The new head CT scan revealed a recurrence of the right subdural hematoma, which was again evacuated through a new burr hole, now placed rostrally, and a closed drainage system (Fig. 2A). In addition, in order to prevent recurrence intraoperatively, the thick neomembranes that were removed and a small amount of CSDH that was entrapped were identified. The neurological status of the patient again completely improved, while the post-operative head CT scan revealed partial hematoma evacuation, which was treated conservatively with steroids [dexamethasone was administered orally in a dose of 80 mg three times a day for 1 week starting at the end of the first post-operative week, and then gradual reduction of the dose (20 mg at a time) every 5 days]. (Fig. 2B). Based on a personalized management, it was decided to administer dexamethasone as an add-on treatment for hematoma recurrence and the option for MMAE was discussed; however, the patient did not attend his regular follow-up.

**Figure 3:**
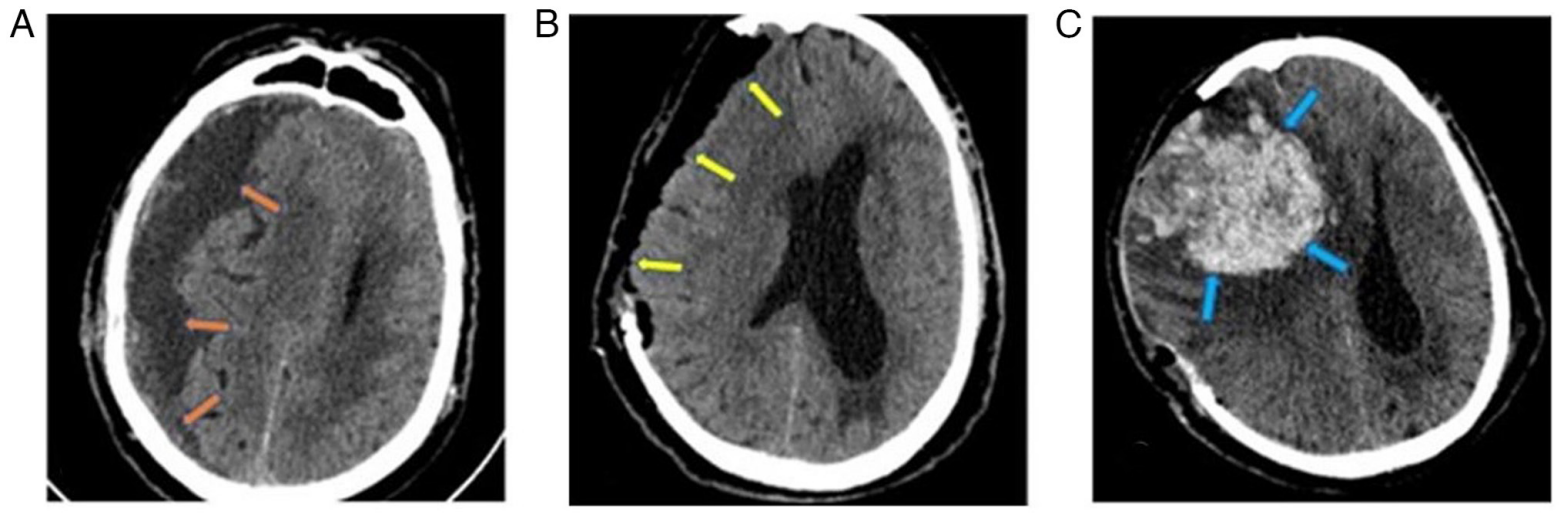
(A) A head CT scan at 2 months after the initial diagnosis revealed a second hematoma recurrence (orange arrows) with a significant mass effect. (B) The patient underwent craniectomy, membranectomy and hematoma evacuation (yellow arrows). (C) The CT scan on the 2nd post-operative day revealed a massive intraparenchymal hemorrhage (blue arrows) and significant edema. CT, computed tomography.

**Figure 4:**
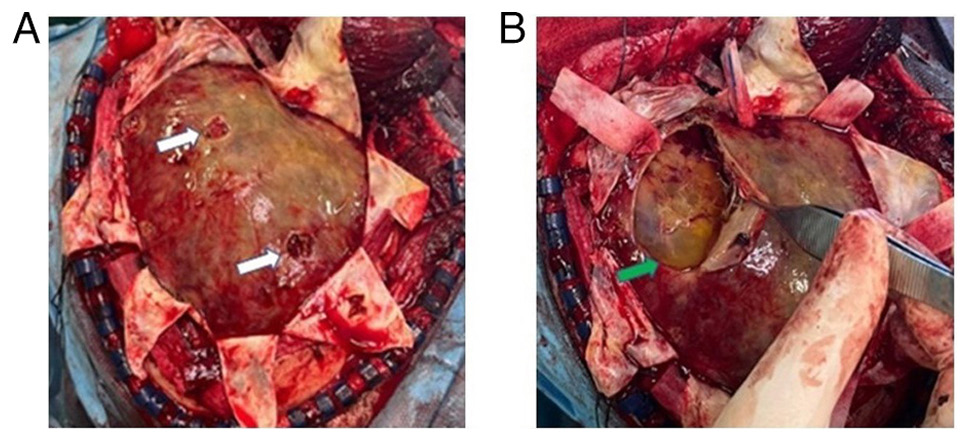
(A) After opening the dura, thick neomembranes appeared to occupy the subdural space. The white arrows show the sites of the two previous craniostomy. (B) The thick neomembranes contained a small amount of yellowish fluid in cavities (green arrow) with septa and trabeculae.

Subsequently, 1 month later, the patient was admitted to the Emergency Room of the University Hospital of Larissa comatose with a GCS score of 6/15 (motor response, 4; verbal response, 1; eye-opening, 1). A new head CT scan revealed a large recurrent subdural hematoma with a significant midline shift (1.45 cm) (Fig. 3A). Considering the history of the patient, he underwent a decompressive craniectomy for hematoma removal. After opening the dura, multiple layers of hard neomembranes trapping a small amount of yellowish fluid in numerous pockets were found (Fig. 4). Therefore, the neomembranes were removed and appropriate hemostasis was performed, followed by layer-by-layer surgical wound closure. An immediate post-operative CT scan revealed complete hematoma removal and midline shift improvement (0.8 cm) (Fig. 3B), and the patient was transferred to the intensive care unit for gradual awakening.

On the 2nd post-operative day, the patient exhibited anisocoria (right, 5 mm; left, 2 mm), which soon changed to fixed-and-dilated pupils and a tense skin flap of the craniectomy. The final head CT scan revealed an extensive intraparenchymal hemorrhage on the right side with a midline shift of 2 cm and a trapped ipsilateral ventricle (Fig. 3C). At the request of the legal representative of the patient, no further surgical intervention was performed, and the patient succumbed within 48 h.

## Discussion

The present case report demonstrates that a relatively benign lesion, such as CSDH, may occasionally exhibit very malignant behavior despite adequate treatment. The malignant nature of CSDH in the case described herein became apparent with the repeated recurrences, the subsequent intraparenchymal hemorrhage, and eventually, the demise of the patient. It is essential to re-consider several clinical and radiological parameters throughout the disease course to identify the ‘point of no return’, if any.

### Risk factors

Zhu *et al* (12) performed a network meta-analysis on the patient-related risk factors that are associated with an increased risk of hematoma recurrence. The patient had several of these predictors. Epidemiological risk factors included an advanced age [standardized mean difference, 0.10; 95% confidence interval (CI), 0.01-0.18], the male sex [relative risk (RR), 1.32; 95% CI, 1.50-1.51] and bilateral location (1.41; 1.20-1.67) (12). The radiological characteristics of the original hematoma did not warn of an increased risk of recurrence, as it was a type 1 lesion (hypodense; RR, 0.79; 0.59-1.05) and not a type 2 lesion (hyperdense, laminar, separated and graded) (12).

### Primary management

Neurosurgeons may opt between single burr hole craniostomy (BHC), double BHC, twist drill craniostomy (TDC) and minicraniotomy to remove CSDH; however, each of these has its own recurrence and reoperation profiles (12,14). There is evidence to suggest that double BHC is the most effective approach (12). In addition, the recurrence rate after BHC has been shown to be lower than after minicraniotomy [odds ratio (OR), 0.58; 95% CI, 0.35-0.97] (15). Another meta-analysis by Yagnik *et al* (14) revealed no difference in the recurrence rate between BHC and TDC (OR, 1.16; 95% CI, 0.84-1.62); however, TDC was associated with a higher reoperation rate, particularly when negative suction drainage was not used (14). Zhu *et al* (12) highlighted the importance of intraoperative saline irrigation (RR, 0.35; 95% CI, 0.19-0.63) and the use of a closed drainage system (RR, 0.45; 0.33-0.60) in reducing the risk of hematoma recurrence. In the patient in the present study, a single BHC was used with warm saline irrigation and a closed drainage system on both sides. Han *et al* (23) reported that the lack of brain re-expansion was the strongest predictor of hematoma recurrence (OR, 25.91; 95% CI, 7.11-94.35) and was strongly associated with senile brain atrophy (OR, 2.36; 1.36-4.11) (23,24). However, the post-operative head CT scan revealed that the brain of the patient never re-expanded sufficiently.

### Management of the first recurrence

According to Henry *et al* (13), when the symptoms recurred, he hematoma was more complex, with a width >20 mm (OR, 2.37; 95% CI, 1.56-3.60) and a midline shift >10 mm (OR, 1.61; 95% CI, 1.17-2.22). Therefore, it was decided to re-evacuate the hematoma through a second BHC, which was connected to a closed drainage system.

Steroids, statins and their combinations have been proposed as medical adjuncts to reduce hematoma recurrence (17,25). The effect of statins and steroids is hypothesized to be mediated by their immunomodulatory properties (25). According to Zhu *et al* (12), patients receiving atorvastatin (OR, 0.31; 95% CI, 0.14-0.69) and corticosteroids (OR 0.41; 95% CI, 0.24-0.70) had a lower hematoma recurrence rate. On the contrary, Monteiro *et al* (16), based on a meta-analysis of seven studies, found insufficient evidence to recommend the regular use of statins (OR, 0.8; 95% CI, 0.35-1.81) in CSDH. In two previous meta-analyses of 12 and five trials, respectively, the authors reported a lower recurrence rate with steroids (OR, 0.39; 95% CI, 0.19-0.79 and RR, 0.4; 95% CI, 0.28-0.58), but at a higher incidence of adverse events (RR, 2.7; 95% CI, 1.71-4.28), including psychiatric symptoms (RR, 3.22; 95% CI, 1.83-5.64), and no difference in neurological outcomes (RR, 1; 95% CI, 0.93-1.08), infection rate (RR, 1.86; 95% CI, 0.56-6.14) and all-cause mortality (RR, 0.66; 95% CI, 0.2-2.18) (26,27). Considering all the available evidence, based on a personalized management, in the present study, it was decided to administer dexamethasone as an add-on treatment for hematoma recurrence and discussed the option for MMAE.

MMAE is considered to reduce the risk of recurrence by interrupting blood supply to the dura, thereby minimizing leakage through the high permeability neomembranes (18,19). It can be used in patients with previously untreated CSDH (upfront MMAE), following surgical hematoma evacuation in cases without any evidence of recurrence (prophylactic MMAE), and for recurrent CSDH after prior surgical excision (18,19). Currently, there is insufficient evidence to indicate that MMAE reduces the risk of recurrence (18,19).

### Management of the second recurrence

In the present study, in the second recurrence, the poor neurological status, significant mass effect, and the failure of previous attempts mandated an urgent decompressive craniectomy. Intraoperatively, the thick neomembranes that were removed and a small amount of CSDH that was entrapped were identified. The role of membranectomy, both inner and outer, or only outer, is still debated (22). An outer membranectomy allows for the uninhibited expansion of the brain by eliminating the mass effect from the neomembranes and reducing the risk of re-bleeding, while inner membranectomy allows for the unimpeded circulation of cerebrospinal fluid through dural lymphatics (22). Hacıyakupoğlu *et al* (22) used craniotomy and membranectomy to treat 13 patients with recurrent CSDH with good results and no recurrence after 3 months.

Endoscopically-assisted burr hole hematoma evacuation offers a safe and effective alternative to craniotomy (20,21,28). A flexible neuroendoscope is inserted through one or two burr holes in the frontal and/or occipital regions (28). Under direct vision, the trabeculae are transected, the compartments of the hematoma cavity are united, and the contents are flushed out with body-warm saline (28). If microhemorrhages occur, a bipolar microcatheter is used for hemostasis (28). Any residual hematoma is drained through a closed-tube system (28). There is recent evidence to indicate that neuroendoscopy reduces recurrence rates compared with conventional treatment (13.1 vs. 3.1% with plain BHC, P<0.001); however, the mortality, morbidity and functional rates remain relatively unaltered (21). In addition, the need for special instruments and training in the use of neuroendoscopy seems to limit its broad usage, as in the case described herein.

### Intraparenchymal hemorrhage

The patient in the present study experienced a massive intraparenchymal hemorrhage (IPH) with a fatal outcome on the 2nd post-operative day. IPH is a rare, yet serious complication following CSDH evacuation (29). In a previous literature review by Krueger *et al* (29), 48 cases were described. IPH frequently occurs in males (85%), with CSDH causing a significant midline shift (54%), at ~1.9 days (±3 days) after surgery (29). The hemorrhage is usually located in the hemisphere ipsilateral (P=0.02) to the hematoma (29). Several interrelated mechanisms, including altered venous circulation, rapid re-expansion of the brain and local edema, have been implicated in the pathogenesis of hemorrhage (29). A second intervention is required in ~27% of cases, with mortality rates reaching as high as 25% (29).

### Point of no return

Table I estimates the probability of hematoma recurrence for each of the aforementioned parameters. OR values were derived from the literature, and the relevant reference citations are cited in the last column. The probability of recurrence was calculated based on the OR values. The most influential factor in the patient described herein was the lack of brain re-expansion (recurrence probability, 96%), followed by hematoma width (70%) and the presence of senile atrophy (70%). The first modifiable factor, the type of surgical evacuation, ranked seventh (54%). Retrospectively, it appears that the craniocerebral mismatch determined the serial recurrences and, as a result, the fate of the patient in the present case report. In theory, MMAE could reduce the risk of recurrence to 13%. However, the available evidence was derived from studies on primary CSDH without focusing on high-recurrence-risk patients, as in the case described herein. The efficacy of MMAE remains to be determined in this population in future studies.

In conclusion, contrary to the common belief, the management of a CSDH is a complex and challenging task. Furthermore, despite advances in the primary treatment of CSDH, the outcomes remain suboptimal in certain cases. Therefore, the literature has extensively explored potential predictors of treatment failure. Recurrent CSDH, characterized by multiple compartments and thick neomembranes, poses even greater challenges. In fact, effective treatment alternatives for such cases are extremely limited, as highlighted herein, including the patient in the present study. Consequently, a more individualized approach is required, which may involve more aggressive treatment options such as craniectomy and membranectomy. In addition, future research is required with the use of more advanced approaches, such as endovascular embolization of the meningeal artery, which may reduce the recurrence rate and lead to improved outcomes in patients with CSDH.

## Figures and Tables

**Table I:** Summary table of the characteristics of the patient in the present study according to the evidence on hematoma recurrence from the literature.

Authors	Parameter	Odds ratio	Probability of recurrence (%)	Modifiable factor	(Refs.)
Han *et al*	Failure of brain re-expansion	25.0	96	No	(23)
Zhu *et al*	Hematoma width (>20 mm)	2.37	70	No	(12)
Han *et al*	Senile brain atrophy	2.36	70	No	(23)
Zhu *et al*	Midline shift (>10 mm)	1.61	62	No	(12)
Zhu *et al*	Bilateral location	1.41	59	No	(12)
Zhu *et al*	Male gender	1.32	57	No	(12)
Yagnik *et al*	Single burr hole	1.16	54	Yes	(14)
Zhu *et al*	Type 1 lesion^a^	0.79	44	No	(12)
Zhu *et al*	Closed drainage system	0.45	41	Yes	(12)
Shrestha *et al*	Steroids	0.39	28	Yes	(26)
Zhu *et al*	Irrigation	0.35	26	Yes	(12)
Ironside *et al*, Jumah *et al*	No MMAE	6.66	87	Yes	(18,19)

^a^In anticipation of <10% baseline risk, it is hypothesized that the odds ratio equals the relative risk. Odds ratios were derived from the literature, and the relevant references are cited in the last column. The probability of recurrence was calculated based on the odds ratio. MMAE, middle meningeal artery embolization.

## Data Availability

The datasets used and/or analyzed during the current study are available from the corresponding author on reasonable request.
